# Causal associations between circulating inflammatory cytokines and blinding eye diseases: a bidirectional Mendelian randomization analysis

**DOI:** 10.3389/fnagi.2024.1324651

**Published:** 2024-01-23

**Authors:** Menghao Teng, Jiachen Wang, Xiaochen Su, Ye Tian, Xiaomin Ye, Yingang Zhang

**Affiliations:** ^1^Department of Orthopedics, The First Affiliated Hospital of Xi'an Jiaotong University, Xi'an, China; ^2^Department of Joint Surgery, HongHui Hospital, Xi'an Jiaotong University, Xi'an, China; ^3^Healthy Food Evaluation Research Center, West China School of Public Health and West China Fourth Hospital, Sichuan University, Chengdu, China; ^4^School of Electronic Information and Artiffcial Intelligence, Shaanxi University of Science and Technology, Xi'an, China

**Keywords:** circulating inflammatory cytokines, glaucoma, cataract, macular degeneration, mendelian randomization, genome-wide association study

## Abstract

**Background:**

Previous studies have explored the associations between circulating inflammatory cytokines and blinding eye diseases, including glaucoma, cataract and macular degeneration. However, the causality of these associations remains controversial. This study employs a bidirectional Mendelian randomization (MR) study to investigate the causal relationships between 41 circulating inflammatory cytokines and these blinding eye diseases.

**Methods:**

Summary data for glaucoma, cataract, macular degeneration and 41 circulating inflammatory cytokines were publicly available. The inverse variance weighted (IVW) method was employed as the main analysis method. Additionally, various sensitivity tests, including MR–Egger regression, weighted median, weight mode, Cochran’s Q test, MR pleiotropy Residual Sum and Outlier test, and leave-one-out test, were conducted to evaluate sensitivity and stability of results.

**Results:**

The IVW analysis identified six circulating inflammatory cytokines causally associated with the risk of blinding eye diseases: Monokine induced by interferon-gamma (MIG) for glaucoma, interleukin-1 receptor antagonist (IL-1ra), IL-6, IL-10, and platelet derived growth factor BB (PDGFbb) for cataract, and MIG and hepatocyte growth factor (HGF) for macular degeneration. However, it is noteworthy that none of these associations remained significant after Bonferroni correction (*p* < 0.0004). Reverse MR analyses indicated that cataract may lead to a decrease in vascular endothelial growth factor (VEGF) levels (OR: 3.326 × 10^−04^, 95% CI: 5.198 × 10^−07^ − 2.129 × 10^−01^, *p* = 0.0151).

**Conclusion:**

This study highlights the potential roles of specific inflammatory cytokines in the development of glaucoma, cataract and macular degeneration. Moreover, it suggests that VEGF is likely to be involved in cataract development downstream. These findings offer insights for early prevention and novel therapeutic strategies for these blinding eye diseases.

## 1 Introduction

Blinding eye diseases have a profound impact on both ocular structure and function, leading to severe visual impairment and, in some cases, complete vision loss, particularly among the elderly population. Among these diseases, glaucoma, cataracts, and macular degeneration stand out as some of the most prevalent and challenging issues in public health worldwide, significantly diminishing the quality of life for those affected and placing substantial burdens on healthcare systems (Liu et al., 2017; Fleckenstein et al., 2021; Kang and Tanna, 2021). Notably, as of 2013, the global prevalence of glaucoma among individuals aged 40 to 80 years reached 3.54%, and it is projected to affect a staggering 111.8 million people by 2040 (Tham et al., 2014). Moreover, in 2020, cataracts affected 17.2% of the populations in various age groups (Hashemi et al., 2020), while macular degeneration affected 7.36% of the Asian population and 9.39% of the white population aged 40 to 79 (Kawasaki et al., 2010). Although extensive attention in epidemiological research, the precise pathogenesis of these blinding eye diseases remains elusive, making it the central focus of ongoing research.

Inflammation, a fundamental component of the immune response, plays a pivotal role in various physiological and pathological processes throughout the body (Kang et al., 2014; Talaat et al., 2015; Singh et al., 2021). Dysregulation of immune effector cells can trigger a systemic inflammatory response, causing damage to target tissues and contributing to the initiation and progression of diseases (Weinstein et al., 2021). Previous studies have underscored a significant correlation between inflammatory cytokines and cataracts, glaucoma and macular degeneration. Patients with cataracts exhibit notable increases in the expression levels of interleukin-6 (IL-6), IL − 1β, and tumor necrosis factor-α (TNF-α) compared to healthy individuals (Dong et al., 2019). Similarly, elevated levels of IL-5, IL-12, IL-15, interferon-gamma (IFN-γ), and monocyte inflammatory protein-1α (MIP-1α) have been detected in the aqueous humor of glaucoma patients (Burgos-Blasco et al., 2020). Additionally, macular degeneration is associated with increasing levels of vascular endothelial growth factor (VEGF), monocyte chemotactic protein-1 (MCP-1), IL-6, and IL-8 in the aqueous humor (Mimura et al., 2019). Nonetheless, an ongoing debate persists regarding whether systemic inflammation acts as a causal factor for cataract, glaucoma and macular degeneration or if it emerges as a consequence of disease progression and medication usage following the onset of these ocular conditions. Consequently, further genetic evidence is warranted to explore the causality between circulating inflammatory cytokines and these blinding eye diseases.

Mendelian randomization (MR) is a valuable epidemiological research technique for dissecting the mechanisms behind disease occurrence (Xu et al., 2020). Single-nucleotide polymorphisms (SNPs) serve as instrumental variables (IVs) to probe the causal association between exposure phenotypes and disease outcomes in MR analysis. Given that genetic variants are randomly assigned at conception and remain impervious to environmental factors, MR can mitigate the influences of confounding factors (Smith and Ebrahim, 2004; Zhou et al., 2019). In addition, MR helps counteract bias stemming from reverse causality since an individual’s genotype is determined at conception and remains unaltered by disease progression (Zhou et al., 2019; Zhao et al., 2022). In this study, we initiated the selection of valid IVs from publicly available summary data originating from a genome-wide association (GWAS) study encompassing 41 circulating inflammatory cytokines (Ahola-Olli et al., 2017). Our primary objective was to assess their associations with cataracts, glaucoma, and macular degeneration. Subsequently, we conducted a deeper investigation by reversing the exposure and outcome variables to elucidate the direction of causation.

## 2 Materials and methods

### 2.1 Study design

**Figure 1:**
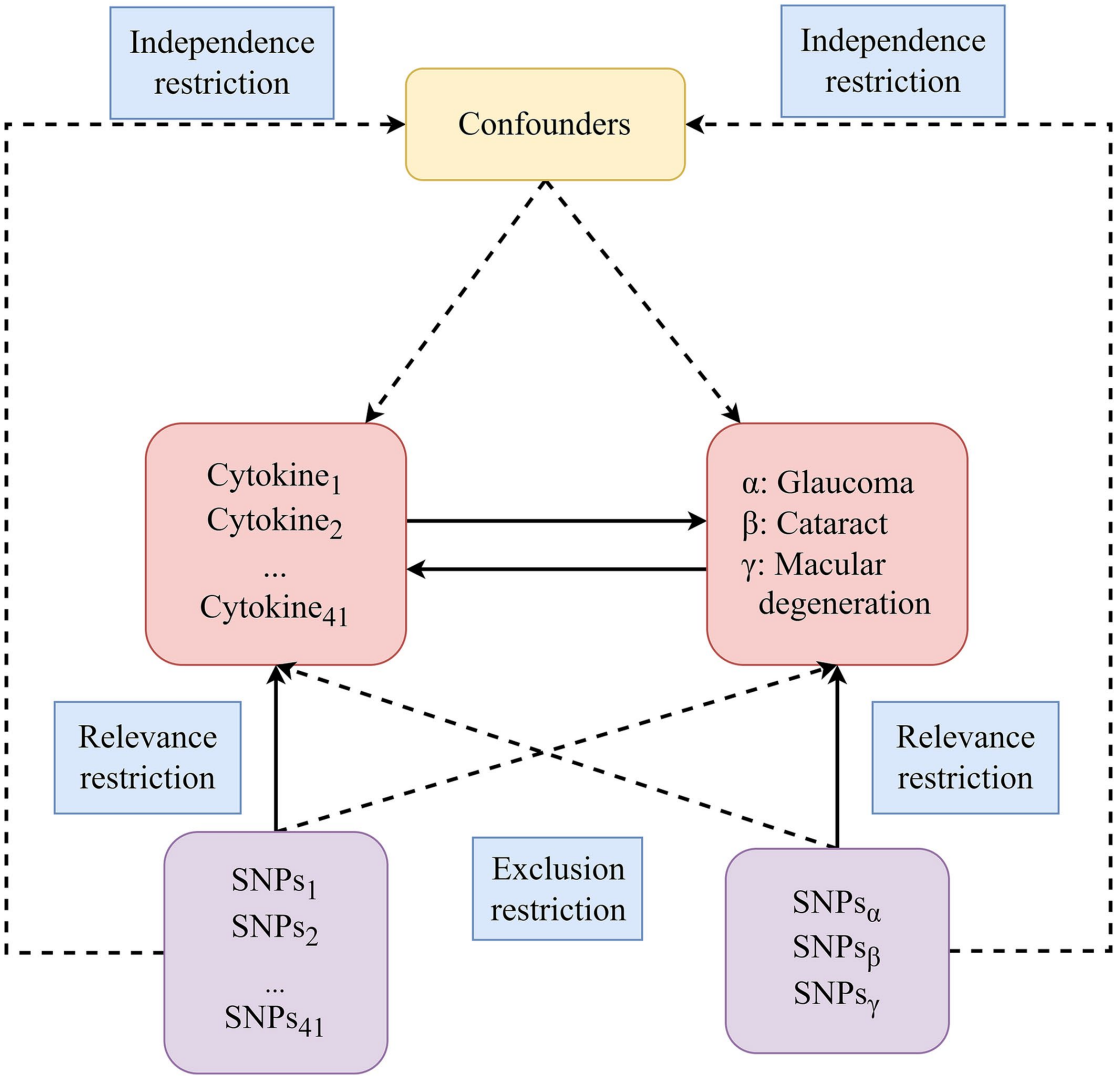
The diagram of the study design in this bidirectional MR analysis. We meticulously identified significant IVs for 41 circulating inflammatory cytokines and three blinding eye diseases, subsequently delving into the examination of bidirectional causal causalities. Three key assumptions of MR analysis were illustrated in this figure, including relevance restriction, independence restriction and exclusion restriction. MR, Mendelian randomization; SNPs, Single-nucleotide polymorphisms.

We utilized publicly available GWAS summary statistics to conduct this bidirectional two-sample MR analysis. Our selection of SNPs as IVs for MR analysis was guided by three key assumptions (illustrated in Figure 1): (a) Relevance restriction: selected SNPs used as IVs must exhibit a robust association with the exposures; (b) Independence restriction: these SNPs should remain independent of confounding factors related to the exposure and outcome; (c) Exclusion restriction: there should be no alternative biological pathway from the genetic instrument to the outcome, apart from the pathway through the exposure.

### 2.2 Data sources for 41 circulating inflammatory cytokines

The summary statistics for 41 circulating inflammatory cytokines used in this study were sourced from a comprehensive large-scale GWAS meta-analysis. This meta-analysis included 8,293 participants from three separate population cohorts: the Cardiovascular Risk in Young Finns Study (YFS, mean age for men: 37.4 years; for women: 37.5 years), FINRISK 1997 (mean age for men: 48.3 years; for women: 47.3 years) and FINRISK 2002 (mean age for men: 60.4 years; for women: 60.1 years) (Ahola-Olli et al., 2017). Cytokines and growth factors were quantified using various sample types, including EDTA plasma in FINRISK 1997, heparin plasma in FINRISK 2002, and serum in YFS. The genetic associations were meticulously adjusted for age, gender, body mass index and the first 10 genetic principal components. The sample size for each circulating inflammatory cytokine analyzed could be found in Supplementary Table S1.

### 2.3 Data sources for glaucoma, cataract and macular degeneration

Summary statistics for glaucoma, cataract and macular degeneration were obtained from the IEU Open GWAS project.^1^ The glaucoma GWAS dataset (ID: ukb-d-H40) encompassed 361,194 individuals, comprising 1715 cases and 359,479 controls, with a total of 10,233,350 SNPs. Similarly, the cataract GWAS dataset (ID: ukb-a-529) included 337,199 individuals, with 2,651 cases and 334,548 controls, and encompassed 10,894,596 SNPs. Finally, the macular degeneration GWAS dataset (ID: ukb-b-17194) involved 150,642 individuals, including 3,553 cases and 147,089 controls, and featured a total of 9,851,867 SNPs. Detailed information regarding the summary data for cataract, glaucoma and macular degeneration used in this study can be found in Supplementary Table S2.

### 2.4 Selection of IVs

SNPs were meticulously selected as IVs from the exposure data for MR analysis. Initially, we selected SNPs strongly associated with exposures at the genome-wide significance threshold (*p* < 5 × 10^−8^). However, for most circulating inflammatory cytokines, we did not find a sufficient number of SNPs meeting this threshold. Therefore, we adopted a higher threshold value (*p* < 5 × 10^−6^) to identify relevant SNPs for circulating inflammatory cytokines. To guarantee the independence of the selected IVs, we applied a stringent clumping process with *r*^2^ < 0.001 and a window size = 10,000 kb to exclude SNPs with strong linkage disequilibrium. Furthermore, to assess the strength of the selected IVs, the F statistic for each SNP was calculated using the formula: $F=\beta^{2}/{\mathrm{SE}}^{2}$ , where β represents the beta for the SNP-exposure association and SE indicates the standard error for the SNP-exposure association (Bowden et al., 2016b). SNPs with *F* statistics<10 were considered weak IVs and were excluded for further MR analysis. Subsequently, we utilized the PhenoScanner V2 website^2^ to filter out SNPs associated with the outcome and potential confounding factors. The PhenoScanner V2 is a comprehensive database that compiles the associations between human genotypes and phenotypes. All well-established risk factors for cataract, glaucoma and macular degeneration, including aging, smoking, alcohol consumption and diabetes mellitus, were considered as potential confounders in our study (Lee and Mackey, 2022; Yuan et al., 2022). Finally, we harmonized the exposure and outcome data, removing palindromic SNPs with intermediate allele frequencies to ensure that the effect alleles matched consistently. In the reverse MR analyses, the selection procedures for IVs from the summary data of glaucoma, cataract and macular degeneration were consistent with those for circulating inflammatory cytokines. All well-known risk factors affecting circulating inflammatory cytokines levels, such as infection and autoimmune diseases, were deemed as potential confounding factors in the reverse MR analysis and were excluded using the PhenoScanner V2 website (Takeuchi and Akira, 2010; Schwartz et al., 2016).

### 2.5 Mendelian randomization analysis

In this study, we employed the inverse variance weighted (IVW) method as the primary analysis method to evaluate causal associations between 41 circulating inflammatory cytokines and blinding eye diseases. The IVW method employs the inverse variances of IVs as weights for a weight calculation to assess the cumulative causal effect (Bowden et al., 2016a). The IVW method operates under the assumption that all selected IVs are valid and provides the most accurate results in the absence of horizontal pleiotropy and heterogeneity (Burgess et al., 2016). Furthermore, in cases where the MR estimate involved only a single SNP, we used the Wald ratio method instead of the IVW method to assess causality (Hartwig et al., 2016).

### 2.6 Sensitivity analysis

First, we employed the mRnd website^3^ to evaluate the statistical power of IVW results, which was based on a non-centrality parameter method, following the method outlined by a previous study (Brion et al., 2013). The sample size in the summary data, odds ratios (OR), and *R*^2^ (variance of exposure explained by each IV) were required for power calculations, and the *R*^2^ for each SNP was calculated using the formula: $R^{2}=2\times \mathrm{EAF}\times \left(1-\mathrm{EAF}\right)\times \beta^{2}$ , where EAF represents the effect allele frequency and *β* is the beta for the SNP-exposure association. Moreover, to further enhance the credibility of our IVW analysis results, we conducted a variety of sensitivity tests, including MR–Egger regression, the weighted median method, the weighted mode method, Cochran’s Q test, the MR Pleiotropy Residual Sum and Outlier (MR-PRESSO) test, and leave-one-out test. MR–Egger regression, capable of furnishing a consistent causal estimate even when all genetic IVs are invalid (Bowden et al., 2015), is of particular interest due to its ability to assess horizontal pleiotropy through the intercept value. The weighted median method can yield relatively precise results, even in scenarios where up to 50% of IVs are invalid (Bowden et al., 2016a). In addition, the weighted mode method was utilized as a supplementary method to further evaluate the reliability of IVW results. Given the susceptibility of these methods to bias from outlying genetic variants and their capacity to offer relatively precise causal estimates in the presence of horizontal pleiotropy and heterogeneity, we prioritized focusing on the direction and effect size of these results in this study (Luo et al., 2022; Yin et al., 2022). Furthermore, Cochran’s Q test was used to detect potential heterogeneity by IVW and MR–Egger methods. Subsequently, the MR-PRESSO global test was utilized to investigate the presence of horizontal pleiotropy. Additionally, we employed the MR-PRESSO outlier test and MR-PRESSO distortion test to identify outliers in the associations and correct for horizontal pleiotropy after excluding these outliers (Verbanck et al., 2018). Furthermore, we performed additional MR analyses after excluding outliers to assess the stability of causal associations. Finally, the leave-one-out sensitivity test was conducted, excluding individual SNP one at a time, to evaluate the stability and validity of causal effect estimates.

We applied the Bonferroni method to address the issue of multiple testing, taking into account the number of circulating inflammatory cytokines. Results with a *p* value below 0.0004 (0.05/123) were regarded as strong evidence, indicating robust statistical significance. Associations that were initially significant (*p* < 0.05) but did not maintain significance following Bonferroni correction (*p* > 0.0004) were considered potential. All statistical analyses were two-sided and performed using R (version 4.2.3) software with the R packages “Two-sample MR,” “forest plot” and “MRPRESSO.”

## 3 Results

### 3.1 Selection of IVs and overview

**Figure 2:**
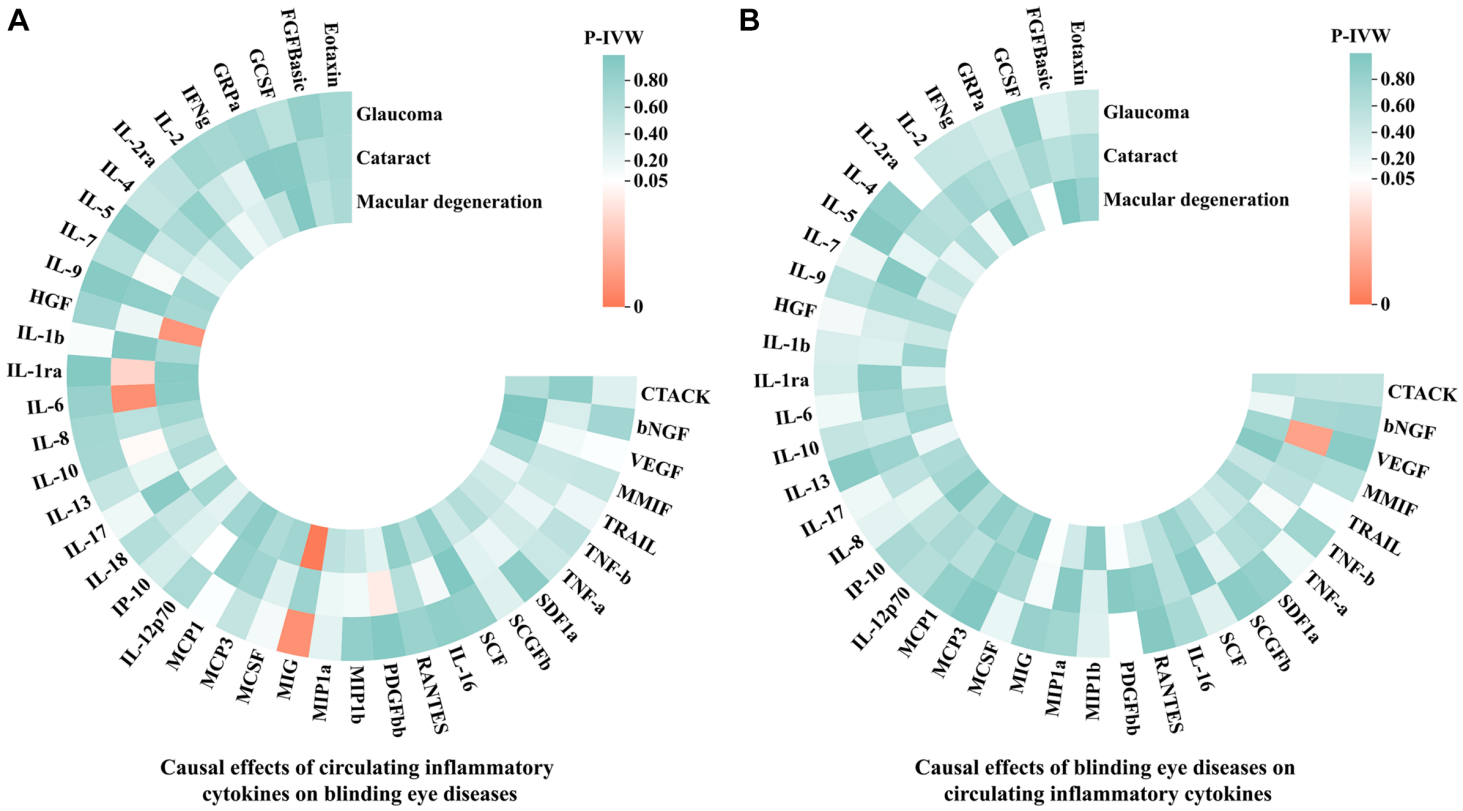
The IVW results of causal associations among 41 circulating inflammatory cytokines and the three blinding eye diseases. **(A)** Causal effects of circulating inflammatory cytokines on blinding eye diseases. **(B)** Causal effects of blinding eye diseases on circulating inflammatory cytokines. IVW, Inverse variance weighted; CTACK, Cutaneous T-cell attracting chemokine; bNGF, Beta-nerve growth factor; VEGF, Vascular endothelial growth factor; MMIF, Macrophage migration inhibitory factor; TRAIL, TNF-related apoptosis-inducing ligand; TNF-b, Tumor necrosis factor beta; TNF-a, Tumor necrosis factor alpha; SDF1a, Stromal-cell-derived factor 1 alpha; SCGFb, Stem cell growth factor beta; SCF, Stem cell factor; IL, Interleukin; RANTES, Regulated on activation, normal T cell expressed and secreted; PDGFbb, Platelet-derived growth factor BB; MIP1b, Macrophage inflammatory protein 1 beta; MIP1a, Macrophage inflammatory protein 1 alpha; MIG, Monokine induced by gamma interferon; MCSF, Macrophage colony stimulating factor; MCP, Monocyte chemoattractant protein; IP-10, Interferon gamma-induced protein 10; HGF, Hepatocyte growth factor; IFNg, Interferon gamma; GRPa, Growth-regulated protein alpha; GCSF, Granulocyte-colony stimulating factor; FGFBasic, Fibroblast growth factor basic levels.

In this MR analysis, we initially set the genome-wide significance threshold value at 5 × 10^−8^. However, for glaucoma, cataract, macular degeneration and the most of circulating inflammatory cytokines, we found an insufficient number of SNPs meeting this threshold. Therefore, we adopted a higher threshold (*p* < 5 × 10^−6^) to ensure an adequate number of SNPs for MR analysis. After meticulously selecting SNPs that exhibited genome-wide independence (*r*^2^ < 0.001, clumping window size = 10,000 kb) and were statistically significant (*p* < 5 × 10^−6^), the number of IVs for circulating inflammatory cytokines and three blinding eye diseases ranged from 4 to 23. More details regarding each SNP are accessible in Supplementary Table S3. Moreover, the lowest F statistic among these SNPs was 11.1597, suggesting that causal associations were not disturbed by weak instrumental bias. Subsequently, the PhenoScanner V2 website revealed that rs13278062, rs1333040, rs635634, and rs10761731 were associated with macular degeneration, glaucoma, diabetes mellitus, and drinking, respectively. These SNPs were excluded from further analysis. Moreover, in the reverse MR analyses, no SNP was excluded at this step. Finally, we aligned the exposure and outcome data to eliminate palindromic SNPs with intermediate allele frequencies, and all SNPs removed during this step were documented in Supplementary Table S4. After this rigorous selection process, the number of IVs ranged from 1 to 21 for circulating inflammatory cytokines and the three blinding eye diseases. Detailed information on all selected SNPs for 41 circulating inflammatory cytokines and the three blinding eye diseases is listed in Supplementary Tables S5, S6, respectively. The overall analysis results by the IVW method are presented in Figure 2.

### 3.2 The causal effects of genetically determined circulating inflammatory cytokines on the risk of glaucoma, cataract and macular degeneration

**Figure 3:**
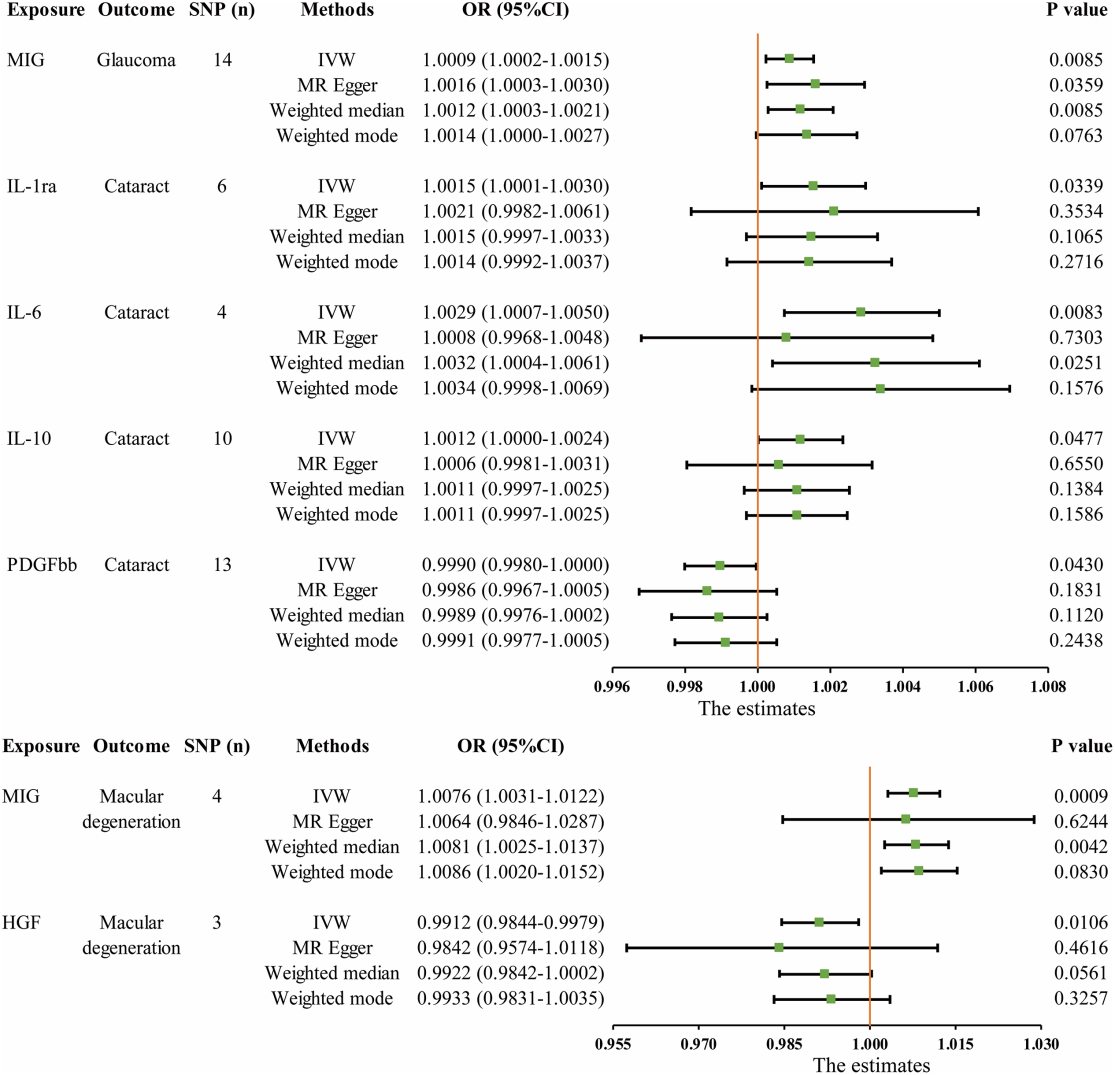
Initially significant associations (*p* < 0.05) between circulating inflammatory cytokines with glaucoma, cataract, and macular degeneration. SNP (n), The number of single-nucleotide polymorphisms; OR, Odds ratios; CI, Confidence interval; IVW, Inverse variance weighted; MIG, Monokine induced by gamma interferon; IL, Interleukin; PDGFbb, Platelet-derived growth factor BB; HGF, Hepatocyte growth factor.

**Figure 4:**
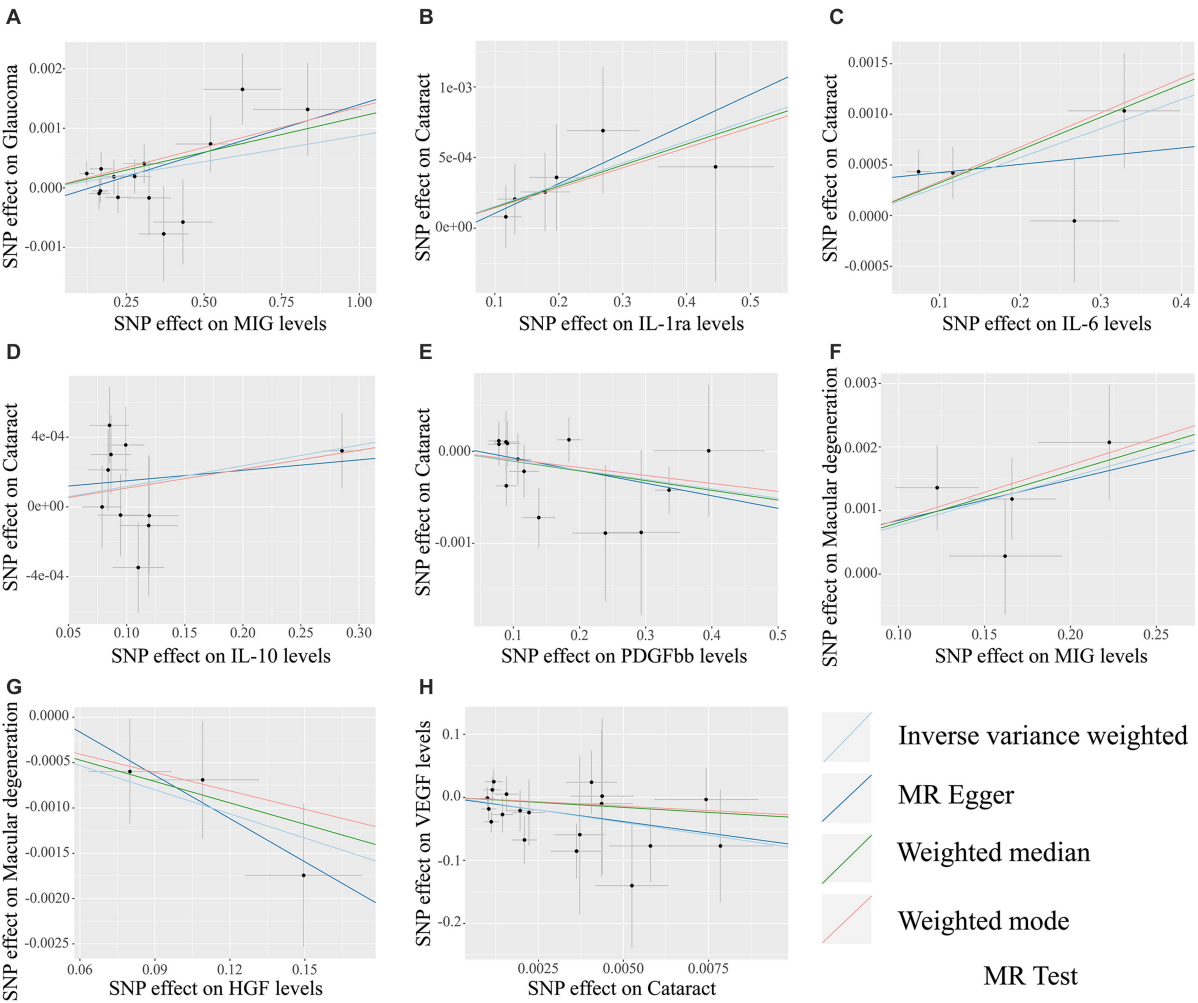
The scatter plots for potential associations (p < 0.05) between circulating inflammatory cytokines and blinding eye diseases by the IVW method. The vertical axis in scatter plots represents the effect of selected SNPs on the outcome, and the horizontal axis is the effect of selected SNPs on the exposure. The causal effect of each analysis method is indicated by the slope of the line. **(A)** MIG on glaucoma. **(B)** IL-1ra on cataract. **(C)** IL-6 on cataract. **(D)** IL-10 on cataract. **(E)** PDGFbb on cataract. **(F)** MIG on macular degeneration. **(G)** HGF on macular degeneration. **(H)** Cataract on VEGF. SNPs, Single-nucleotide polymorphisms; MIG, Monokine induced by gamma interferon; IL, Interleukin; PDGFbb, Platelet-derived growth factor BB; HGF, Hepatocyte growth factor; VEGF, Vascular endothelial growth factor.

The MR analysis results concerning the causality between 41 circulating inflammatory cytokines and the risk of glaucoma, cataract and macular degeneration are presented in Figure 3 and Supplementary Table S7. The IVW results revealed several noteworthy associations. Monokine induced by interferon-gamma (MIG) exhibited a potential association with an increasing risk of glaucoma (OR: 1.0009, 95% CI: 1.0002–1.0015, *p* = 0.0085). Furthermore, interleukin-1 receptor antagonist (IL-1ra, OR: 1.0015, 95% CI: 1.0001–1.0030, *p* = 0.0339), IL-6 (OR: 1.0029, 95% CI: 1.0007–1.0050, *p* = 0.0083), and IL-10 (OR: 1.0012, 95% CI: 1.0000–1.0024, *p* = 0.0477) were potentially associated with an elevated risk of cataract, while platelet derived growth factor BB (PDGFbb) appeared to be a potential protective factor for cataract (OR: 0.9990, 95% CI: 0.9980–1.0000, *p* = 0.0430). Additionally, MIG (OR: 1.0076, 95% CI: 1.0031–1.0122, *p* = 0.0009) and hepatocyte growth factor (HGF, OR: 0.9912, 95% CI: 0.9844–0.9979, *p* = 0.0106) were associated with the risk of macular degeneration. Notably, it is mentioned that none of these associations remained significant after applying a Bonferroni correction (*p* < 0.0004). The scatter plots provided a more intuitive visualization for initially significant results (*p* < 0.05) in Figures 4A–G.

### 3.3 The causal effects of blinding eye diseases on circulating inflammatory cytokines levels

**Figure 5:**
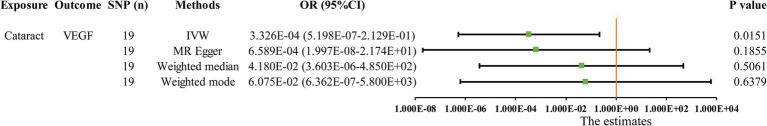
Initially significant association (*p* < 0.05) between three blinding eye diseases with circulating inflammatory cytokines, as determined by the IVW method. SNP (n), The number of single-nucleotide polymorphisms; OR, Odds ratios; CI, Confidence interval; IVW, Inverse variance weighted; VEGF, Vascular endothelial growth factor.

The MR analysis results for the influence of glaucoma, cataract and macular degeneration on circulating inflammatory cytokines levels are presented in Figure 5 and Supplementary Table S8. It was found by the IVW results that cataract was associated with a reduced level of vascular endothelial growth factor (VEGF, OR: 3.326 × 10^−04^, 95% CI: 5.198 × 10^−07^-2.129 × 10^−01^, *p* = 0. 0151). The scatter plot provided a more intuitive visualization for the causality between cataract and VEGF levels in Figure 4H.

### 3.4 Sensitivity analysis

The statistical power of our IVW results, detailed in Supplementary Table 9, was somewhat limited due to small sample sizes (Brion et al., 2013; Burgess, 2014). In addition, MR–Egger regression, the weighted median method, and the weighted mode method either supported the results by IVW or pointed toward a similar directional trend, which enhanced the reliability of our analysis results. Additionally, Table 1 displays the results of heterogeneity and horizontal pleiotropy for all initially significant associations (*p* < 0.05) between 41 circulating inflammatory cytokines and three blinding eye diseases. Cochran’s Q test revealed the absence of heterogeneity in these associations (all *p* > 0.05). Additionally, both the Egger intercept and MR-PRESSO global test indicated no evidence of horizontal pleiotropy in any of the associations (all *p* > 0.05). The MR-PRESSO outlier test did not identify any outliers within these associations. The distribution of IVs in funnel plots, as depicted in Supplementary Figure S1, was symmetric, further suggesting the absence of outliers and horizontal pleiotropy in these potential associations. Finally, the leave-one-out test demonstrated that excluding any single SNP did not influence these causal associations, confirming the stability and reliability of our results, as shown in Supplementary Figure S2.

**Table 1:** The results of sensitivity tests for all initially significant associations (*p* < 0.05) between 41 circulating inflammatory cytokines and three blinding eye diseases.

Exposure	Outcome	Cochran’s Q test		Egger Intercept test		MR-PRESSO	
		Q-IVW	*p*-value	Egger-intercept	*p*-value	*p*-value of global test	*p*-value of distortion test
MIG	Glaucoma	13.184	0.434	-2.15E-04	0.242	0.101	NA
IL-1ra	Cataract	0.712	0.982	-1.06E-04	0.776	0.877	NA
IL-6	Cataract	3.005	0.391	3.44E-04	0.358	0.574	NA
IL-10	Cataract	9.755	0.371	8.94E-05	0.621	0.557	NA
PDGFbb	Cataract	10.369	0.584	6.41E-05	0.681	0.702	NA
HGF	Macular degeneration	0.498	0.780	7.92E-04	0.698	0.476	NA
MIG	Macular degeneration	1.680	0.641	2.05E-04	0.922	0.741	NA
Cataract	VEGF	15.717	0.612	-1.80E-03	0.871	0.440	NA

IVW, Inverse variance weighted; MR-PRESSO, MR Pleiotropy RESidual Sum and Outlier; MIG, Monokine induced by gamma interferon; IL, Interleukin; PDGFbb, Platelet-derived growth factor BB; HGF, Hepatocyte growth factor; VEGF, Vascular endothelial growth factor.

## 4 Discussion

We conducted a bidirectional two-sample MR analysis to assess causal associations among three blinding eye diseases and 41 circulating inflammatory cytokines. The MR analysis genetically predicted that the levels of six distinct circulating inflammatory cytokines might be causally linked to the risk of these blinding eye diseases, including MIG for glaucoma, IL-1ra, IL-6, IL-10 and PDGFbb for cataract, and MIG and HGF for macular degeneration. In the reverse MR analyses, we found that cataract leads to a decrease in VEGF levels. Notably, there was no reverse causality between a single circulating inflammatory cytokine and these blinding eye diseases. These findings suggest that several certain circulating inflammatory cytokines may play a crucial role in initiating the development of glaucoma, cataract and macular degeneration, while VEGF is more likely to lie downstream during cataract progression.

Glaucoma, cataract, and macular degeneration stand out as the most prevalent age-related eye diseases, intricately linked to the aging process. The aging process triggers various pathological events within the retina, encompassing the activation of endothelial cells, free radical-induced oxidative damage, and heightened concentrations of inflammatory cytokines (Xu et al., 2009). Notably, these events culminate in the activation of the NOD-, LRR-, and pyrin domain-containing protein 3 inflammasome (Maran et al., 2023), further contributing to retinal ganglion cells (RGCs) apoptosis, lens epithelial cells (LECs) damage, lens opacification, lipofuscin accumulation in retinal pigment epithelial (RPE) cells, and the formation and accumulation of drusen, ultimately leading to the development of glaucoma, cataracts, and macular degeneration (Liu and Zhu, 2017; Alqawlaq et al., 2019; Maran et al., 2023). In recent years, the exploration of antioxidants, such as polyphenols, vitamins, and carotenoids, has unveiled their potential to counteract oxidative stress and slow disease progression in the early stages of glaucoma, cataracts, and macular degeneration (Giannaccare et al., 2020; Zhang et al., 2020; Maiuolo et al., 2022). Simultaneously, the role of inflammation in the development and progression of these eye diseases has garnered increased attention (Lim et al., 2020). Elevated levels of inflammatory cytokines have been observed in the eyes of patients with these conditions (Ten Berge et al., 2019). Moreover, another study highlighted the potential influence of a pro-inflammatory diet in promoting the onset of these blinding eye diseases (Vergroesen et al., 2023). In summary, inflammatory factors play a pivotal role in the development of these three blinding eye diseases. Therefore, the early identification of high-risk individuals through the recognition of inflammatory factors is crucial for health management in the elderly population and reducing the societal burden.

Glaucoma is a neurodegenerative disorder characterized by the progressive loss of RGCs, degeneration and degradation of optic nerve axons, and optic disk atrophy (Kwong et al., 2013; Cohen and Pasquale, 2014). Previous studies have established a robust molecular foundation for the role of inflammation in the pathogenesis of glaucoma (Baudouin et al., 2021). Increased intra-ocular pressure has been demonstrated to trigger elevated levels of pro-inflammatory cytokines, such as IL-1, IL-6, IL-8, and TNF-α (Alvarado et al., 2015; Cueva Vargas et al., 2015). These cytokines, in a cascading sequence, activate nitric oxide synthase expression (Yuan and Neufeld, 2000), induce mitochondrial dysfunction (Kaur et al., 2013), modulate endothelial permeability and stimulate the matrix metalloproteinases expression (Liton et al., 2005). These multifaceted effects eventually culminate in the death of RGCs and axonal degeneration, ultimately contributing to the onset of glaucoma (Roh et al., 2012). Furthermore, individuals diagnosed with glaucoma have been observed to exhibit elevated levels of various inflammatory cytokines, including IL-9, IL-10, IL-12, IFN-α, and MIG (Chua et al., 2012). Consistent with these previous findings, our study has identified a potential association between elevated MIG levels and an increased risk of glaucoma. MIG, belonging to the CXC cytokine family and induced by IFN-γ, plays a critical role in immune cell chemotaxis and the recruitment of cells expressing CXCR3 receptors (Ouedraogo et al., 2013; Tokunaga et al., 2018). Notably, MIG goes further by promoting the polarization of effector Th cells via CXCR3 receptors, thereby reinforcing the immune response and subsequently elevating the expression of TNF-α, IL-2 and IFN-γ (Mosser and Edwards, 2008; Tokunaga et al., 2018). This intricate interplay leads to the death of RGCs, axonal degeneration, and the progression of glaucoma. Our findings underscore the complex relationship between MIG and glaucoma, revealing its potential as a key mediator in the pathogenic cascade of this vision-threatening disease.

Several previous studies have reported elevated levels of various ILs in patients with cataract (Chen et al., 2014; Sauer et al., 2016; Jin et al., 2018; Dong et al., 2019; Engelbrecht et al., 2020). Consistent with these findings, our results found that IL-1ra, IL-6 and IL-10 levels were potentially associated with the increased risk of cataract. IL-1ra, a natural anti-inflammatory agent and inflammatory marker, belongs to the IL-1 family and can bind to IL-1 receptors, thereby blocking the pro-inflammatory effects of IL-1α and IL-1β (Hotamisligil, 2017). In recent years, IL-1ra has been proposed for the treatment of various systemic and local inflammatory pathologies. However, IL-1ra has also been shown to upregulate before the onset of many inflammatory diseases, such as type 2 diabetes mellitus and psoriasis, which may be related to the early compensatory response to inflammation caused by IL-1 and TNF (Herder et al., 2009; Kim et al., 2016). IL-10, a cytokine with multiple immunosuppressive functions, affects antigen presentation, release of immune mediators, and phagocytosis in monocytes/macrophages. Additionally, IL-10 inhibits the expression of MHC class II and co-stimulatory molecules and reduces the production of IL-1β and TNF-α (Sabat, 2010). A previous study observed elevated intraocular IL-10 levels in diabetic cataract patients, which might be a compensatory response to enhanced expression of proinflammatory mediators (Mitrović et al., 2016). Based on this, we speculate that the elevated levels of IL-1ra and IL-10 represent a compensatory response aimed at maintaining body homeostasis in the presence of systemic inflammation. This systemic inflammatory state leads to an increase in reactive oxygen and nitrogen species, eventually resulting in lens opacities and cataracts. IL-6, a pleiotropic cytokine, regulates inflammation and immune responses by interacting with various cell types, including leukocytes, endothelial cells and fibroblasts (Rose-John et al., 2006). Its bioactivity is mediated by a membrane-binding receptor comprising two subunits: a signal transduction molecule glycoprotein 130 (gp130) and a homologous receptor subunit (IL-6R) that specifically recognizes IL-6 (Chen et al., 2016). Previous studies have revealed a correlation between elevated IL-6 levels in serum and advancing age, which is a known risk factor for cataract (Maggio et al., 2006; Forcina et al., 2022). Consequently, we speculate that individuals with elevated IL-6 levels may share a significant overlap with the aging population, which provides a potential explanation for the association between elevated IL-6 levels and an increased risk of cataracts.

Our findings provided genetic evidence supporting the protective role of PDGFbb against cataracts. The LECs, the sole cell type within the lens, play a pivotal role in maintaining the internal environment of the lens, which is a critical factor for ensuring its optical transparency (Huang et al., 2022). PDGFbb, a member of the PDGF family, is involved in various physiological processes, such as mitosis, differentiation, chemotaxis and angiogenesis (Wang et al., 2019). Within the eye, PDGFbb promotes the proliferation of LECs, thus acting as a safeguard against cataract development that may result from a decline in LECs (Li et al., 2019; Paensuwan et al., 2022). Moreover, previous studies have reported elevated levels of VEGF in cataract patients (Mitrović et al., 2016), which is inconsistent with our findings. In cases where patients are preparing for cataract surgery, anti-VEGF drugs are often required to enhance postoperative vision recovery (Zhao and Cheng, 2019; Mehta, 2021). We speculate that the decrease in systemic VEGF levels found in individuals with cataract in our study could be attributed to anti-VEGF treatment. Therefore, further investigation is warranted to fully understand the relationship between VEGF levels and cataracts.

Our study revealed that MIG was a potential risk factor for macular degeneration, aligning with previous studies (Agawa et al., 2014; Spindler et al., 2018). Macular degeneration is a neurodegenerative retinal disease characterized by abnormal neovascularization, degeneration of photoreceptors, and RPE cells dysfunction (Askou, 2014). The RPE cells in the eye serve various vital functions, crucial for the maintenance of neural cells, rods, and cones, preserving retinal health, and maintaining visual function (Strauss, 2005; Kaarniranta et al., 2011). Dysfunction and inflammation in RPE cells are vital factors to contribute to macular degeneration and the subsequent loss of vision (Ambati et al., 2013). MIG, classified as a pro-inflammatory chemokine, promotes the release of TNF-α, IL-2, and IFN-γ in effector Th cells by binding to CXCR3 receptors (Tokunaga et al., 2018). These inflammatory mediators play a vital role in mediating inflammatory responses and dysfunction in RPE cells, ultimately contributing to the development of macular degeneration (Kutty et al., 2013; Mirra et al., 2022). HGF, a pleiotropic growth factor, is produced by various ocular cells, including the RPE, pericytes, fibroblasts, iris epithelium, lens epithelium and trabecular cells (Li et al., 1996; Hu and Ritch, 2001). HGF plays a crucial role in responding to injury and can stimulate the growth and migration of a variety of ocular cells, including RPE, iris epithelium, lens epithelium, and vascular endothelium cells (He et al., 1998; Lashkari et al., 1999; Hu and Ritch, 2001; Jun et al., 2007). In addition, Kouji et al. reported a protective effect of HGF on RPE cells against degeneration in sodium iodate-injected rats (Ohtaka et al., 2006). Our study further supports these previous studies, highlighting HGF as a protective factor in the development of macular degeneration.

To our knowledge, this is the first MR study to explore causal associations among 41 circulating inflammatory cytokines and three blinding eye diseases, including glaucoma, cataract and macular degeneration. Our study offers several advantages. First, the usage of the MR method minimizes the influence of bias caused by confounders and reverse causality. We obtained SNPs from various large GWAS summary data, and all SNPs used in this study underwent rigorous selection. Moreover, the lowest F statistic among selected IVs in our study was 11.1597, indicating a minimal possibility of weak instrumental bias influencing the results. Finally, we employed various methods to evaluate the sensitivity of all initially significant associations, ensuring the stability of MR analysis results. However, it is crucial to acknowledge several limitations in the current MR study. First, the observed relatively low statistical power in IVW results and the restricted number of IVs for specific associations, such as the one between MIG and macular degeneration, may be attributed to the limited sample size within the available database (Brion et al., 2013; Burgess, 2014). Therefore, future research efforts should encompass larger GWAS dataset to validate these conclusions. In addition, our summary data were exclusively sourced from individuals of European ancestry, necessitating caution when applying our findings to other populations. Furthermore, the inherent nature of GWAS summary data precluded access to detailed clinical information about the cases, which in turn hindered further subgroup analysis. Lastly, we must recognize that our study did not comprehensively investigate the underlying pathogenesis of circulating inflammatory factors leading to the development of three blinding eye diseases. Further research in this area is imperative for a more thorough understanding in the future.

## 5 Conclusion

This bidirectional MR analysis has successfully pinpointed several potential associations among three blinding eye diseases and 41 circulating inflammatory cytokines. Specifically, we identified one upstream circulating inflammatory cytokine linked to glaucoma, four upstream circulating inflammatory cytokines along with one downstream effector associated with cataract, and two upstream circulating inflammatory cytokines correlated with macular degeneration. These findings hold promise for early prevention strategies and the development of novel therapeutics for these blinding eye diseases. However, it is imperative that additional research is conducted to validate the precise roles of specific circulating inflammatory cytokines in the pathogenesis of these blinding eye diseases, leading to a deeper understanding of the underlying disease mechanisms.

## 6 Limitations of the study

Our study acknowledges several potential limitations that warrant consideration: (1) Statistical power: the study’s statistical power is constrained by the limited sample size within the utilized database, leading to cautious interpretation of IVW results; (2) Ethnicity restriction: the exclusive reliance on summary data from individuals of specific ethnic backgrounds, primarily of European ancestry, raises concerns about generalizability to diverse populations; (3) Lack of subgroup analyses: the absence of detailed clinical information impedes the execution of subgroup analyses, limiting a more nuanced exploration of potential associations; (4) Insufficient exploration of pathogenesis: the study provides valuable associations but falls short of a comprehensive exploration of the underlying pathogenesis of the observed associations. Each limitation has been thoroughly discussed in the text to provide transparency and context to our study’s constraints. Furthermore, considering that these associations stem from silico analyses, additional validation through rigorous animal studies and population-based research is essential for robust confirmation.

## Data availability statement

The original contributions presented in the study are included in the article/Supplementary material, further inquiries can be directed to the corresponding author.

## Author contributions

MT: Conceptualization, Formal analysis, Writing – original draft, Writing – review & editing. JW: Conceptualization, Formal analysis, Writing – original draft, Writing – review & editing. XS: Investigation, Writing – original draft. YT: Investigation, Writing – original draft. XY: Software, Writing – original draft. YZ: Project administration, Writing – review & editing.

## Footnotes

^1^http://gwas.mrcieu.ac.uk/

^2^www.phenoscanner.medschl.cam.ac.uk

^3^https://shiny.cnsgenomics.com/mRnd/
