# Supplementary material for: Causal associations between circulating inflammatory cytokines and blinding eye diseases: a bidirectional Mendelian randomization analysis
Source: Front Aging Neurosci. 2024 Jan 23;16:1324651. doi: 10.3389/fnagi.2024.1324651 (PMC10848324; doi:10.3389/fnagi.2024.1324651)
Supplement: Supplementary file 2 [file Table_2.DOCX]

Supplementary Material

**Causal associations between circulating inflammatory cytokines and blinding eye diseases: a bidirectional Mendelian randomization analysis**

**Menghao Teng, Jiachen Wang, Xiaochen Su, Ye Tian, Jiqing Wang, Xiaomin Ye, Yingang Zhang^*^**

***** **Correspondence:** Corresponding Author E-mail: [zyingang@mail.xjtu.edu.cn](mailto:zyingang@mail.xjtu.edu.cn)

***Supplementary Tables Legends:***

**Supplementary Table 1.** The sample size for 41 circulating inflammatory cytokines used in this study acquired from the GWAS.

**Supplementary Table 2.** Detailed information on the summary data for glaucoma, cataract and macular degeneration used in this study.

**Supplementary Table 3.** Detailed information on initially selected instrumental variables of 41 circulating inflammatory cytokines and three blinding eye diseases. Note: SNP: Single-nucleotide polymorphisms; Beta: Estimate coefficient; SE: Standard error; EAF: Effect allele frequency; R2: The genetic variation; CTACK: Cutaneous T-cell attracting chemokine; bNGF: Beta-nerve growth factor; VEGF: Vascular endothelial growth factor; MMIF: Macrophage migration inhibitory factor; TRAIL: TNF-related apoptosis-inducing ligand; TNF-b: Tumor necrosis factor beta; TNF-a: Tumor necrosis factor alpha; SDF1a: Stromal-cell-derived factor 1 alpha; SCGFb: Stem cell growth factor beta; SCF: Stem cell factor; IL: Interleukin; RANTES: Regulated on activation, normal T cell expressed and secreted; PDGFbb: Platelet-derived growth factor BB; MIP1b: Macrophage inflammatory protein 1 beta; MIP1a: Macrophage inflammatory protein 1 alpha; MIG: Monokine induced by gamma interferon; MCSF: Macrophage colony stimulating factor; MCP: Monocyte chemoattractant protein; IP-10: Interferon gamma-induced protein 10; HGF: Hepatocyte growth factor; IFNg: Interferon gamma; GRPa: Growth-regulated protein alpha; GCSF: Granulocyte-colony stimulating factor; FGFBasic: Fibroblast growth factor basic levels.

**Supplementary Table 4.** All removed palindromic SNPs with intermediate allele frequencies. Note: SNP: Single-nucleotide polymorphisms; Beta: Estimate coefficient; SE: Standard error; CTACK: Cutaneous T-cell attracting chemokine; bNGF: Beta-nerve growth factor; VEGF: Vascular endothelial growth factor; MMIF: Macrophage migration inhibitory factor; TRAIL: TNF-related apoptosis-inducing ligand; TNF-b: Tumor necrosis factor beta; TNF-a: Tumor necrosis factor alpha; SDF1a: Stromal-cell-derived factor 1 alpha; SCGFb: Stem cell growth factor beta; SCF: Stem cell factor; IL: Interleukin; RANTES: Regulated on activation, normal T cell expressed and secreted; PDGFbb: Platelet-derived growth factor BB; MIP1b: Macrophage inflammatory protein 1 beta; MIP1a: Macrophage inflammatory protein 1 alpha; MIG: Monokine induced by gamma interferon; MCSF: Macrophage colony stimulating factor; MCP: Monocyte chemoattractant protein; IP-10: Interferon gamma-induced protein 10; HGF: Hepatocyte growth factor; IFNg: Interferon gamma; GRPa: Growth-regulated protein alpha; GCSF: Granulocyte-colony stimulating factor; FGFBasic: Fibroblast growth factor basic levels.

**Supplementary Table 5.** Detailed information on instrumental variables of 41 circulating inflammatory cytokines used in MR analyses. Note: SNP: Single-nucleotide polymorphisms; Beta: Estimate coefficient; SE: Standard error; CTACK: Cutaneous T-cell attracting chemokine; bNGF: Beta-nerve growth factor; VEGF: Vascular endothelial growth factor; MMIF: Macrophage migration inhibitory factor; TRAIL: TNF-related apoptosis-inducing ligand; TNF-b: Tumor necrosis factor beta; TNF-a: Tumor necrosis factor alpha; SDF1a: Stromal-cell-derived factor 1 alpha; SCGFb: Stem cell growth factor beta; SCF: Stem cell factor; IL: Interleukin; RANTES: Regulated on activation, normal T cell expressed and secreted; PDGFbb: Platelet-derived growth factor BB; MIP1b: Macrophage inflammatory protein 1 beta; MIP1a: Macrophage inflammatory protein 1 alpha; MIG: Monokine induced by gamma interferon; MCSF: Macrophage colony stimulating factor; MCP: Monocyte chemoattractant protein; IP-10: Interferon gamma-induced protein 10; HGF: Hepatocyte growth factor; IFNg: Interferon gamma; GRPa: Growth-regulated protein alpha; GCSF: Granulocyte-colony stimulating factor; FGFBasic: Fibroblast growth factor basic levels.

**Supplementary Table 6.** Detailed information on instrumental variables of three blinding eye diseases used in MR analyses. Note: SNP: Single-nucleotide polymorphisms; Beta: Estimate coefficient; SE: Standard error; CTACK: Cutaneous T-cell attracting chemokine; bNGF: Beta-nerve growth factor; VEGF: Vascular endothelial growth factor; MMIF: Macrophage migration inhibitory factor; TRAIL: TNF-related apoptosis-inducing ligand; TNF-b: Tumor necrosis factor beta; TNF-a: Tumor necrosis factor alpha; SDF1a: Stromal-cell-derived factor 1 alpha; SCGFb: Stem cell growth factor beta; SCF: Stem cell factor; IL: Interleukin; RANTES: Regulated on activation, normal T cell expressed and secreted; PDGFbb: Platelet-derived growth factor BB; MIP1b: Macrophage inflammatory protein 1 beta; MIP1a: Macrophage inflammatory protein 1 alpha; MIG: Monokine induced by gamma interferon; MCSF: Macrophage colony stimulating factor; MCP: Monocyte chemoattractant protein; IP-10: Interferon gamma-induced protein 10; HGF: Hepatocyte growth factor; IFNg: Interferon gamma; GRPa: Growth-regulated protein alpha; GCSF: Granulocyte-colony stimulating factor; FGFBasic: Fibroblast growth factor basic levels.

**Supplementary Table 7.** The causal estimates of 41 circulating inflammatory cytokines on three blinding eye diseases obtained by four MR methods. Note: SNP (n): The number of single-nucleotide polymorphisms; IVW: Inverse variance weighted; CTACK: Cutaneous T-cell attracting chemokine; bNGF: Beta-nerve growth factor; VEGF: Vascular endothelial growth factor; MMIF: Macrophage migration inhibitory factor; TRAIL: TNF-related apoptosis-inducing ligand; TNF-b: Tumor necrosis factor beta; TNF-a: Tumor necrosis factor alpha; SDF1a: Stromal-cell-derived factor 1 alpha; SCGFb: Stem cell growth factor beta; SCF: Stem cell factor; IL: Interleukin; RANTES: Regulated on activation, normal T cell expressed and secreted; PDGFbb: Platelet-derived growth factor BB; MIP1b: Macrophage inflammatory protein 1 beta; MIP1a: Macrophage inflammatory protein 1 alpha; MIG: Monokine induced by gamma interferon; MCSF: Macrophage colony stimulating factor; MCP: Monocyte chemoattractant protein; IP-10: Interferon gamma-induced protein 10; HGF: Hepatocyte growth factor; IFNg: Interferon gamma; GRPa: Growth-regulated protein alpha; GCSF: Granulocyte-colony stimulating factor; FGFBasic: Fibroblast growth factor basic levels.

**Supplementary Table 8.** The causal estimates of three blinding eye diseases on 41 circulating inflammatory cytokines obtained by four MR methods. Note: SNP (n): The number of single-nucleotide polymorphisms; IVW: Inverse variance weighted; CTACK: Cutaneous T-cell attracting chemokine; bNGF: Beta-nerve growth factor; VEGF: Vascular endothelial growth factor; MMIF: Macrophage migration inhibitory factor; TRAIL: TNF-related apoptosis-inducing ligand; TNF-b: Tumor necrosis factor beta; TNF-a: Tumor necrosis factor alpha; SDF1a: Stromal-cell-derived factor 1 alpha; SCGFb: Stem cell growth factor beta; SCF: Stem cell factor; IL: Interleukin; RANTES: Regulated on activation, normal T cell expressed and secreted; PDGFbb: Platelet-derived growth factor BB; MIP1b: Macrophage inflammatory protein 1 beta; MIP1a: Macrophage inflammatory protein 1 alpha; MIG: Monokine induced by gamma interferon; MCSF: Macrophage colony stimulating factor; MCP: Monocyte chemoattractant protein; IP-10: Interferon gamma-induced protein 10; HGF: Hepatocyte growth factor; IFNg: Interferon gamma; GRPa: Growth-regulated protein alpha; GCSF: Granulocyte-colony stimulating factor; FGFBasic: Fibroblast growth factor basic levels.

**Supplementary Table 9.** The statistical power of IVW analysis results. Note: SNP (n): The number of single-nucleotide polymorphisms; IVW: Inverse variance weighted; CTACK: Cutaneous T-cell attracting chemokine; bNGF: Beta-nerve growth factor; VEGF: Vascular endothelial growth factor; MMIF: Macrophage migration inhibitory factor; TRAIL: TNF-related apoptosis-inducing ligand; TNF-b: Tumor necrosis factor beta; TNF-a: Tumor necrosis factor alpha; SDF1a: Stromal-cell-derived factor 1 alpha; SCGFb: Stem cell growth factor beta; SCF: Stem cell factor; IL: Interleukin; RANTES: Regulated on activation, normal T cell expressed and secreted; PDGFbb: Platelet-derived growth factor BB; MIP1b: Macrophage inflammatory protein 1 beta; MIP1a: Macrophage inflammatory protein 1 alpha; MIG: Monokine induced by gamma interferon; MCSF: Macrophage colony stimulating factor; MCP: Monocyte chemoattractant protein; IP-10: Interferon gamma-induced protein 10; HGF: Hepatocyte growth factor; IFNg: Interferon gamma; GRPa: Growth-regulated protein alpha; GCSF: Granulocyte-colony stimulating factor; FGFBasic: Fibroblast growth factor basic levels.

***Supplementary Figures Legends:***

**Supplementary Figure 1.** Funnel plots for the initially significant associations between 41 circulating inflammatory cytokines and three blinding eye diseases. (A) MIG on glaucoma. (B) IL-1ra on cataract. (C) IL-6 on cataract. (D) IL-10 on cataract. (E) PDGFbb on cataract. (F) MIG on macular degeneration. (G) HGF on macular degeneration. (H) Cataract on VEGF. MIG: Monokine induced by gamma interferon; IL: Interleukin; PDGFbb: Platelet-derived growth factor BB; HGF: Hepatocyte growth factor; VEGF: Vascular endothelial growth factor.

**Supplementary Figure 2.** The leave-one-out sensitivity tests for the initially significant associations between 41 circulating inflammatory cytokines and three blinding eye diseases. (A) MIG on glaucoma. (B) IL-1ra on cataract. (C) IL-6 on cataract. (D) IL-10 on cataract. (E) PDGFbb on cataract. (F) MIG on macular degeneration. (G) HGF on macular degeneration. (H) Cataract on VEGF. MIG: Monokine induced by gamma interferon; IL: Interleukin; PDGFbb: Platelet-derived growth factor BB; HGF: Hepatocyte growth factor; VEGF: Vascular endothelial growth factor.
