# Supplementary figures and images for: Causal associations between circulating inflammatory cytokines and blinding eye diseases: a bidirectional Mendelian randomization analysis
Source: Front Aging Neurosci. 2024 Jan 23;16:1324651. doi: 10.3389/fnagi.2024.1324651 (PMC10848324; doi:10.3389/fnagi.2024.1324651)

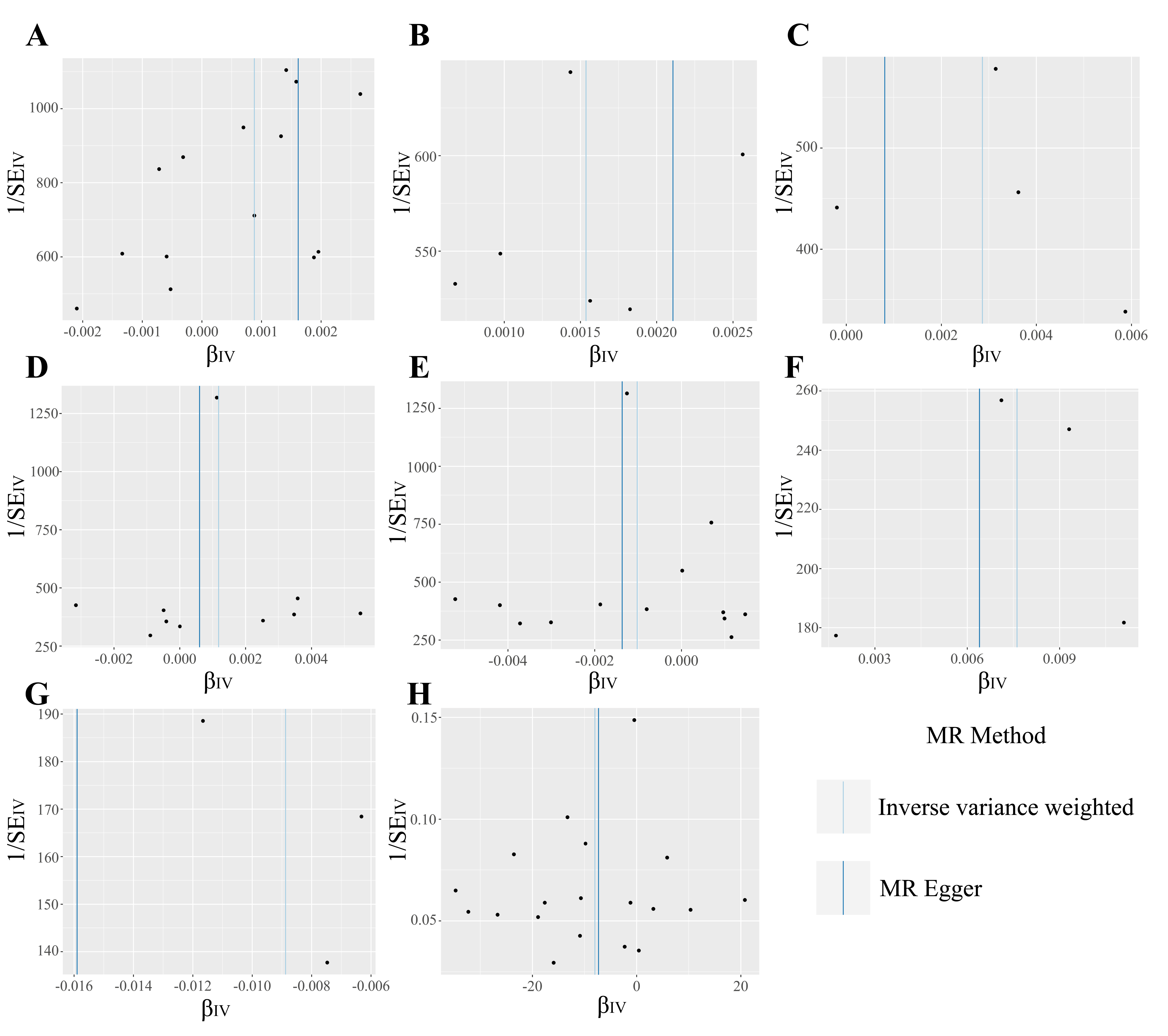

Supplement: Supplementary file 3 [file Image_1.TIF]

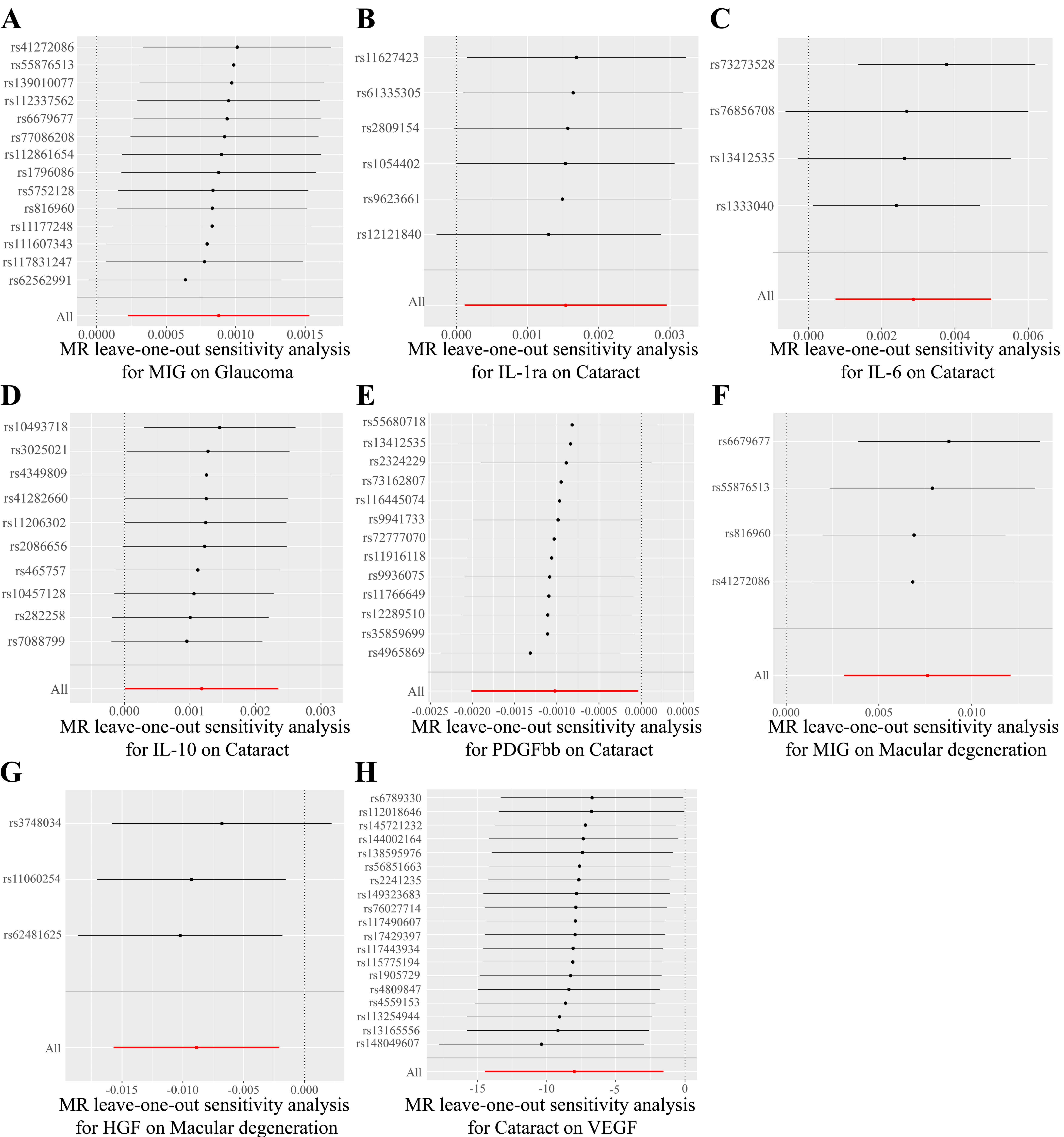

Supplement: Supplementary file 4 [file Image_2.TIF]
